# The Domestic Staff—III

**Published:** 1908-09-12

**Authors:** 


### NURSING ADMINISTRATION.

### THE DOMESTIC STAFF.—III.

### RULES AND REGULATIONS.

THE code of rules which guides the domestic staff must be simple and elastic, but its broad outlines ought to be well defined, for it is in the nature of a contract between mistress and maid. Rules are essential for the protection of the servant fully as much as for the convenience of the employer. The houses where there is no system and where rules are despised as unnecessary are the houses where there is never a moment's leisure, and where every maid grumbles that she is over-worked. It is important that the duties and privileges of the maids should be very clearly understood by them before they are engaged, and the enclosed paper of rules in use at Guy's Hospital is an excellent model.

### RULES FOR DOMESTIC SERVANTS.

TIME TABLE FOR WARDMAIDS.—6 A.M., bell rings; 6.35 A.M., marked on duty; breakfast, 8.30 A.M.; dinner, 1 P.M.; tea, 5.30 P.M.; supper, 8 P.M.; prayers, 8.30 P.M.; in dormitories, 9.30 P.M.; lights out, 10.30 P.M.; after which no talking is allowed. Candles strictly forbidden.

TIMES OFF FOR WARDMAIDS.—One hour each afternoon. Once a week, 2 to 6 P.M. Once a month, 2 to 9.30 P.M. Sundays from 10.30 A.M. to 4.30 P.M.; or 2 to 6 P.M. One Sunday from 2 to 9.30 P.M.

(The table for the home maids varies in one or two particulars.)

Any maid marked late a second time in the week must forfeit her 2 to 6 P.M., and remain on duty. Maids returning to their wards late on duty must forfeit their off-duty time. Those persistently late must lose their half-days.

No maid is allowed to meet her friends at the gates or about the hospital. Permission must be obtained to have visitors. Notes and parcels can be left at the lodge.

The maids are expected to keep themselves neat and tidy; to be quiet and orderly in their behaviour at all times; to be punctual at their meals, and to do everything in their power to promote the comfort and well-being of all.

After three years' service a whole day off duty each month, with breakfast in bed, is allowed. After two years' service a whole day off duty alternate months is given.

The maids are expected to make their beds and keep their cubicles tidy. Six ornaments or photographs are allowed, and nothing else must be left out or a fine will be imposed.

The matron engages and dismisses the domestic servants. Their engagements may be terminated at any time by one month's notice on either side, or by payment of wages in lieu of notice.

It is, of course, understood that the above time-table is merely a skeleton of the day's work, and in no degree supplies the place of the detailed enumeration of each maid's duties, which varies in each instance and is so framed as to inform the housekeeper where each maid is at work and what she is doing during every hour of the day. There is no doubt that the observance of exact rules both in respect of duty and off-duty hours is easier in a large than in a small establishment. There is more formality in the process of marking 50 or 100 maids on duty than when but 10 or 12 are present to answer to the call. It is easier to get the maids off duty punctually when the staff is large enough to supply the places of the absentees without complications ensuing. But if things are to go smoothly an effort must be made even in the smallest cottage hospital to instil a sense of exact punctuality in the service, and to keep faith in regard to off-duty hours, however inconvenient it may be. The matron who is bent on getting and keeping a thoroughly efficient domestic staff, must look beyond the immediate stress of getting the day's work done with as little worry to herself as possible, and study the larger question of inducing her maids to appreciate their position in the household, and to recognise that the situation they are in is likely to advance their future prospects. It will be observed that the off-duty hours for the maids in the above table are afternoon hours. This is a matter of the utmost importance and it is too often neglected. It can lead to nothing but disaster to keep young women confined to the house through the entire daylight hours, and set them free at a time when temptation awaits them either in London or the provincial town. In one of the best-managed provincial nursing homes it has been our privilege to visit the custom is for each maid to get two hours off on three afternoons a week. The staff is not larger than the average, and the work is unquestionably better done than in many establishments where such a privilege would be considered Utopian. But whatever measure of time be granted the principle is the same, to keep a brisk spirit of punctuality stirring right through the establishment, and to let it be perceived that duty and off-duty hours alike are to be observed with exactitude.

The following code of rules has been found to meet various contingencies in the dormitories among young servants:—

### WARD AND DORMITORY MAIDS' RULES.

1. No tea or food of any kind allowed in the cubicles. 2. No flowers or *candles*. 3. All boxes are to be emptied and kept in the boxroom. Nothing is to be kept under the beds; shoes to be put into cupboards. 4. Towels, brush and comb bags, etc., to be kept in the places provided, and anything found lying about will be taken away. 5. Not more than six pictures, including photographs, allowed, and *no nails* are to be knocked into the walls. 6. The maids must strip and make their own beds every morning, draw back their curtains, and leave their cubicles tidy. 7. All rubbish to be put into the white pails provided for that purpose. 8. All drawers and cupboards to be kept locked. any keys found lying about will be given to the sister in charge. 9. No talking after the lights are put out at 10.30. P.M. The maids are requested to behave quietly in the dormitories. Loud talking or laughing on the *stairs* or in the passages is forbidden.

Signed ———, Matron.

Where the number of maids is large the housekeeping sister, or whoever is appointed to supervise the staff, ought to have her room on the same floor, otherwise disorderly conduct at night will inevitably ensue. In small establishments it should suffice to place one of the upper servants in charge, and give her full responsibility for keeping order.

